# Therapeutic interventions to restore microcirculatory perfusion following experimental hemorrhagic shock and fluid resuscitation: A systematic review

**DOI:** 10.1111/micc.12650

**Published:** 2020-08-20

**Authors:** Anoek L. I. van Leeuwen, Nicole A. M. Dekker, Elise P. Jansma, Christa Boer, Charissa E. van den Brom

**Affiliations:** ^1^ Department of Anesthesiology Experimental Laboratory for VItal Signs Amsterdam UMC Vrije Universiteit Amsterdam Cardiovascular Sciences Amsterdam The Netherlands; ^2^ Department of Physiology Amsterdam UMC Vrije Universiteit Amsterdam Cardiovascular Sciences Amsterdam The Netherlands; ^3^ Department of Epidemiology and Biostatistics Amsterdam UMC Vrije Universiteit Amsterdam Public Health research institute Amsterdam The Netherlands; ^4^ Medical Library Vrije Universiteit Amsterdam The Netherlands

**Keywords:** animal models, capillary perfusion, fluid resuscitation, hemorrhagic shock

## Abstract

**Objective:**

Microcirculatory perfusion disturbances following hemorrhagic shock and fluid resuscitation contribute to multiple organ dysfunction and mortality. Standard fluid resuscitation is insufficient to restore microcirculatory perfusion; however, additional therapies are lacking. We conducted a systematic search to provide an overview of potential non‐fluid‐based therapeutic interventions to restore microcirculatory perfusion following hemorrhagic shock.

**Methods:**

A structured search of PubMed, EMBASE, and Cochrane Library was performed in March 2020. Animal studies needed to report at least one parameter of microcirculatory flow (perfusion, red blood cell velocity, functional capillary density).

**Results:**

The search identified 1269 records of which 48 fulfilled all eligibility criteria. In total, 62 drugs were tested of which 29 were able to restore microcirculatory perfusion. Particularly, complement inhibitors (75% of drugs tested successfully restored blood flow), endothelial barrier modulators (100% successful), antioxidants (66% successful), drugs targeting cell metabolism (83% successful), and sex hormones (75% successful) restored microcirculatory perfusion. Other drugs consisted of attenuation of inflammation (100% not successful), vasoactive agents (68% not successful), and steroid hormones (75% not successful).

**Conclusion:**

Improving mitochondrial function, inhibition of complement inhibition, and reducing microvascular leakage via restoration of endothelial barrier function seem beneficial to restore microcirculatory perfusion following hemorrhagic shock and fluid resuscitation.

**Abbreviations**
ALM: adenosine, lidocaine, and magnesium
ATP: adenosine triphosphate
eNOS: endothelial NO synthase
HB/M: a combination of beta‐hydroxybutyrate and melatonin
NO: nitric oxide
PARP: poly(ADP‐ribose) polymerase
PRISMA: Preferred Reporting Items for Systematic Reviews and Meta‐Analyses
RBC: red blood cell
ROS: reactive oxygen species
SYRCLE: Systematic Review Centre for Laboratory Animal Experimentation
TNF‐α: tumor necrosis factor‐α

## 1 INTRODUCTION

Microcirculatory perfusion disturbances following hemorrhagic shock and fluid resuscitation are a major complication and associated with the development of multiple organ dysfunction and increased mortality.^1^ Standard treatment of hemorrhagic shock involves control of bleeding followed by fluid resuscitation. A combination of crystalloids and blood products is given to improve reperfusion and oxygenation of ischemic tissue.^2^ Microcirculatory perfusion is impaired early following hemorrhagic shock^1^, ^3^; however, fluid resuscitation fails to restore microcirculatory perfusion.^3^, ^4^ As microcirculatory perfusion is essential for tissue delivery of oxygen and nutrients, its persistent impairment^3^, ^4^, ^5^ is detrimental for organ function. Therefore, additional therapeutic strategies to restore microcirculatory perfusion are warranted.

The pathophysiology of hemorrhagic shock and fluid resuscitation is complex and involves a systemic inflammatory response, coagulation disturbances, mitochondrial dysfunction, and endothelial activation.^6^ The vascular endothelium plays a key role in the pathophysiology of hemorrhagic shock via the regulation of coagulation, inflammation, leukocyte trafficking, and vascular tone and permeability.^7^ Under normal circumstances, endothelial cells are tightly bound and leakage of fluids to the interstitium is relatively low.^8^ However, during inflammation, as seen during hemorrhagic shock, the endothelium is activated by proinflammatory mediators. This leads to increased endothelial permeability,^7^, ^9^ with progressive leakage of fluids to the interstitium and, eventually, tissue edema. To enable sufficient blood flow, restoration of the circulating volume is necessary; however, fluid resuscitation can also further aggravate fluid leakage and tissue edema.^7^, ^10^ Tissue edema increases diffusion distances between capillaries^11^ leading to impaired tissue perfusion, reduced ATP production, and mitochondrial dysfunction.^12^, ^13^ Blood transfusion, hypothermia, and acidosis are other factors evoking the development of coagulopathy, complement activation,^14^ and systemic inflammation.^7^, ^15^ The systemic inflammatory response is accompanied by the formation of ROS, which are normally neutralized by antioxidants.^12^ However, hemorrhagic shock‐induced mitochondrial dysfunction causes a shift of the balance toward increased ROS production over antioxidant neutralization capacity, which leads to cell apoptosis and tissue damage.^12^

Additional therapeutic interventions are warranted to improve the success rate of the current treatment strategies to restore microcirculatory perfusion following hemorrhagic shock and fluid resuscitation. Hence, the aim of this review was to provide an overview of the effect of non‐fluid‐based therapeutic interventions, given in addition to fluid resuscitation, on microcirculatory perfusion following experimental hemorrhagic shock compared to untreated, with fluid resuscitated controls.

## 2 METHODS

### 2.1 Protocol and registration

This systematic review conforms to the standards of reporting according to the PRISMA reporting guideling^16^ (PRISMA checklist: Table S1) and registered at the International Prospective Register of Systematic Reviews (PROSPERO; CRD42018095432).^17^ During analysis, it appeared that by following the PROSPERO protocol, the extent of extracted data was very large and heterogenic, which made it impossible to give a clear overview. Therefore, in this review only the results of non‐fluid‐based therapeutic agents on microcirculatory perfusion were reported, which deviates from the PROSPERO protocol that includes microcirculatory perfusion as primary outcome and microvascular leakage as secondary outcome.

### 2.2 Eligibility criteria

This systematic review included animal studies with any type, depth, and duration of experimental hemorrhagic shock; however, exchange transfusion was excluded. Any form, volume, and duration of fluid resuscitation with either shed blood, crystalloids, or colloids were included. Any type, dose, and timing of non‐fluid‐based drug administration were included; however, drugs had to be administrated in addition to standard fluid resuscitation. The control population consisted of animals with hemorrhagic shock and solely fluid resuscitation with either shed blood, crystalloids, or colloids. Study protocols with trauma were included, but additional sepsis, pregnancy, aneurysms, or alcohol intoxication was excluded. All outcome parameters reflecting microcirculatory flow dynamics were eligible (eg, flow rate, RBC velocity, or functional capillary density), independent of unit of measures. These parameters reflect the efficiency of microcirculatory perfusion via either the flow rate of RBC per tissue weight (blood flow), flow rate of blood cells solely (RBC velocity), or the distribution of perfused vessels in a certain organ (functional capillary density, number of perfused vessels).

### 2.3 Information sources and search

To identify eligible studies, PubMed, EMBASE.com, and The Cochrane Library (Wiley) electronic bibliographic databases were searched in collaboration with a medical information specialist (EJ and AvL). The first search was run in February 2019. The search was re‐run on March 6, 2020, before final analysis to retrieve most recent studies for inclusion. The full search strategy (Appendix S1) was based on the combination of the following search components: ‘hemorrhagic shock’, ‘capillaries’ or ‘microvasculature’ and ‘perfusion’ or ‘flow velocity’. Reviews, meeting abstracts, conference reports, letters, or editorials were excluded.

### 2.4 Study selection

Screening was performed in two phases: initial screening based on title and abstract followed by full‐text screening of the eligible articles for final inclusion. Titles and abstracts of studies retrieved using the search strategy and those from additional sources were screened independently by two observers (AvL and CvdB). Duplicates were identified and removed using EndNote™ (EndNote X7.4, Thomson Reuters), and studies that potentially met the inclusion criteria were identified according to the above‐mentioned inclusion and exclusion criteria. The screening results were organized in EndNote. The full texts of these potentially eligible studies were retrieved and independently assessed for eligibility by two observers. Discrepancies were resolved through discussion and consensus. The reference lists of included studies were screened for additional eligible studies not retrieved by our search (snowball search).

### 2.5 Data collection

Data extraction was conducted by one reviewer (AvL) and confirmed by another (CvdB). The following data were extracted and assembled in Microsoft Excel:

1. General study characteristics (author, year, country),
2. Animal model (species, age, sex, experimental groups, body weight),
3. Experimental model (hemorrhagic shock protocol: shock induction, target blood pressure, duration of shock; fluid resuscitation protocol: type of resuscitation fluid, volume, duration of follow‐up period),
4. Type of intervention (timing, dosage, route of administration, vehicle),
5. Outcome measurement (technique, organ, and time point of measurement) and outcome measures (quantitative results).

### 2.6 Risk of bias in individual studies

Risk of bias was determined by two independent reviewers (AvL and CvdB), based on the SYRCLE Risk of Bias tool.^18^ Risks of bias were scored for the ten entries as described by Hooijmans et al^18^ and supplemented with two questions addressing treatment of a sham/control group and inclusion of a power calculation. “Yes,” “no,” or “unclear” was used to indicate a low, high, or unclear risk of bias, respectively. Any statement of randomization or blinding was scored with “yes,” and absence of a certain statement was reported as “no.” Disagreements were resolved through consensus‐oriented discussion. Final statements of randomization and blinding at any point were formulated based on the risk assessment tool. The possibility of publication bias was assessed by evaluating a funnel plot of the trial standardized mean differences for asymmetry.

### 2.7 Summary measures and analysis

The intention of the current study is to give an overview of the effect of non‐fluid‐based therapeutic agents on microcirculatory perfusion. We did not limit the target organ or type of measurement, as this would influence outcome. Consequently, the results of the retrieved studies varied widely with regard to outcome parameter, techniques, unit measures, and organ. Due to this, additional quantitative analysis such as a meta‐analysis was not feasible. Our summary measures therefore take the form of a qualitative interpretation and a narrative analysis. Results of individual studies are reported in table format to combine experimental details with outcome measures. Significant differences in means as reported by the authors were categorized as “effective” when a significant increase was reported and “non‐effective” when no statistical effect or a significant decrease in blood flow was reported. Subsequently, studies were classified based on working mechanism. To provide 95% confidence intervals, individual studies are plotted in forest plots (Review Manager 5.3, Cochrane Centre, The Cochrane Collaboration). In case of multiple outcome measurements per study, only blood flow or RBC velocity was used in the descriptive results, with blood flow determined as most valuable. The results regarding characteristics and details of experimental protocol are reported as percentage of the total amount of studies. For the results regarding the microvascular flow assessment, the results are reported as number per working mechanism and percentage of effective studies per working mechanism.

## 3 RESULTS

### 3.1 Study selection

**Figure 1:**
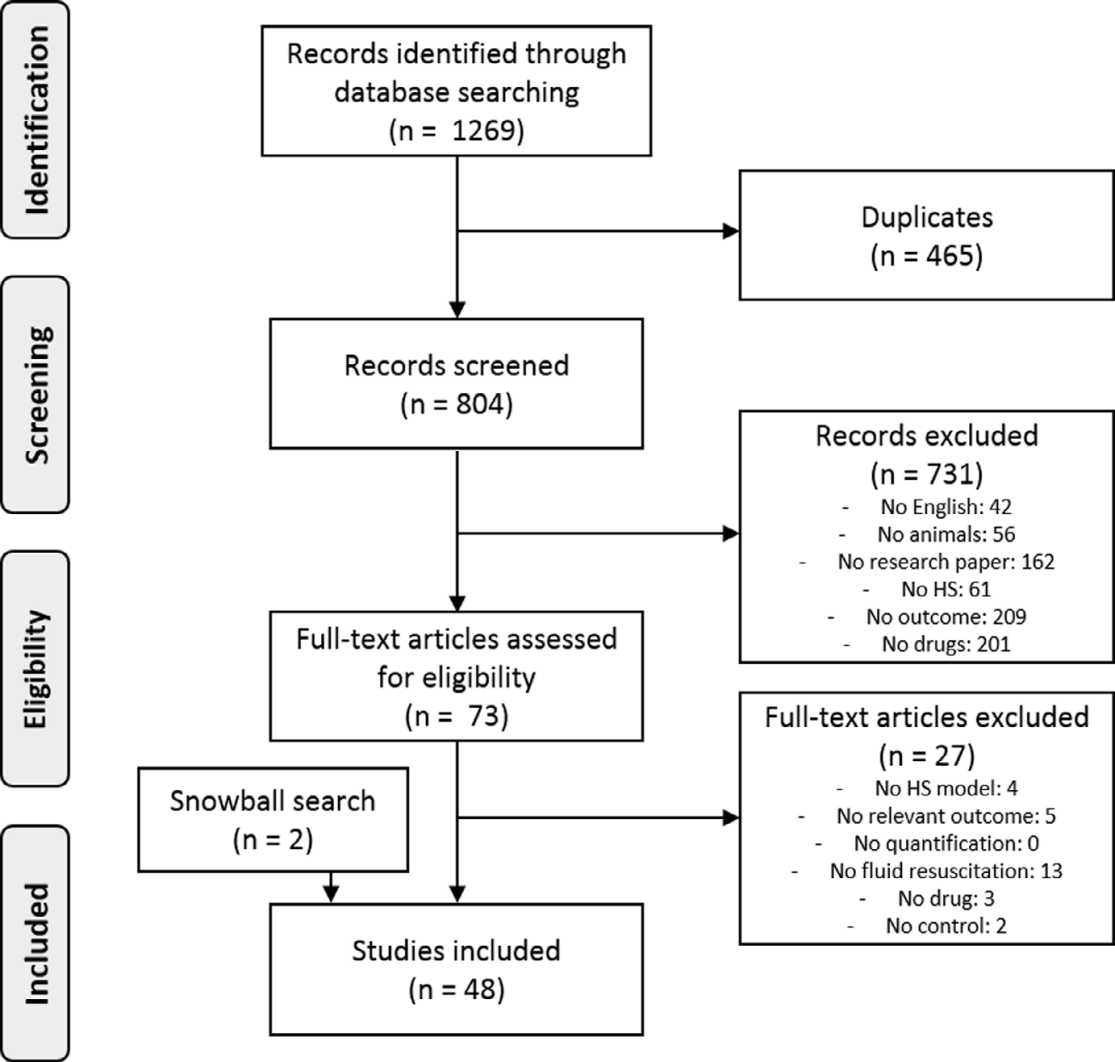
PRISMA diagram representing the flowchart of study selection. PRISMA, Preferred Reporting Items for Systematic Reviews and Meta‐Analyses

The search strategy is presented in a PRISMA diagram (Figure 1). The systematic literature search yielded 1269 records. After removal of duplicates (n = 465), 804 records were screened from which 73 full texts were subsequently examined for eligibility. Two additional records were identified through a snowball search. Finally, 48 studies were included, published between 1969 and 2019, by 35 individual authors. Countries of origin are listed as follows: Austria (n = 1), Brazil (n = 3), Canada (n = 3), China (n = 1), Germany (n = 11), Hungary (n = 1), Italy (n = 2), Japan (n = 1), the Netherlands (n = 1), the UK (n = 1), and the United States (n = 23).

### 3.2 Study characteristics

Study characteristics of all included studies are presented in Table 1. Studies were performed in four different species, with rats being mainly used (77%). Other species used were hamsters (13%), dogs (8%), and pigs (2%). Most studies (71%) used male animals, seven studies (15%) used female animals, six studies (12%) did not report the sex, and one study (2%) explicitly stated the use of both sexes. The number of used animals varied from 4 to 26 animals per group.

**Table 1:** Study characteristics and hemorrhagic shock protocols

Species	Studies (number)	Sex (male/ female/ both/ ND)	Group size (number)	Weight range (g)	Hemorrhagic shock protocol				Resuscitation protocol		References
					Pressure/ volume controlled	Target MAP (mm Hg)	Duration HS (min)	Trauma (number)	Resuscitation fluid (number)	Follow‐up (min)	
Rat	37	28/ 6/ 0/ 3	4‐26	150‐390	35/ 2	25‐65	15‐120	18	Blood (4), crystalloid (17), Blood + crystalloid (16)	30‐24 h	(13, 19, 21, 22, 23, 24, 25, 26, 27, 28, 30, 31, 32, 33, 34, 35, 36, 37, 38, 39, 40, 41, 42, 43, 45, 49, 50, 51, 52, 53, 55, 58, 61, 62, 63, 64, 65
Dog	4	0/ 1/ 1/ 2	5‐15	15 500‐36000	3/ 1	30‐50	60‐210	2	Blood (2), colloid (1), Blood + crystalloid (1)	60‐180	(46, 47, 48, 59
Hamster	6	6/ 0/ 0/ 0	5‐10	55‐140	3/ 3	30‐60	45‐60	1	Blood (3), crystalloid (2), colloid (1)	60‐120	(29, 44, 54, 56, 57, 60
Pig	1	0/ 0/ 0/ 1	10	8000‐12 000	0/ 1	50	ND	0	Crystalloid (1)	ND	(20)

Note

Study characteristics of included studies.

Abbreviations: HS, hemorrhagic shock; MAP, mean arterial pressure; ND, not determined.

### 3.3 Experimental protocol

Details regarding the used experimental models are presented in Table 1. Most studies (85%) used a fixed‐pressure model to induce hemorrhage. Target mean arterial pressure varied from 25 to 65 mm Hg. The duration of the shock period ranged from 15 minutes to 3.5 hours. Three studies did not report the duration of the shock period.^19^, ^20^, ^21^ In approximately half the studies, trauma was induced (44%), mainly by a midline laparotomy.

Resuscitation fluid consisted of crystalloids (42%), a combination of crystalloids and blood (35%), blood only (19%), or colloids (4%). Overall, the volume of fluids was based on the volume withdrawn blood to induce hemorrhagic shock and ranged from 25% to 500% of the volume blood withdrawn. Follow‐up time after start of fluid resuscitation ranged from 30 minutes to 24 hours. Two studies did not report their follow‐up time.^20^, ^22^

### 3.4 Quality assessment

None of the studies met all SYRCLE criteria, indicating a risk of bias for all studies. The majority of the studies (58%) reported randomization at any point and only 25% reported blinding at any point. Half of the studies reported similar baseline values; the remaining studies showed differences at baseline or did not report baseline values. Individual results of the quality assessment per study (Table S2) and a summary of the total quality assessment (Figure S1) are provided as supplemental data. There was a potential risk of publication bias as the funnel plot of all studies revealed asymmetry (Figure S2).

### 3.5 Microvascular flow assessment

The non‐fluid‐based therapeutic interventions were grouped based on their general working mechanisms, resulting in the following groups: antioxidants (n = 7), drugs targeting cell metabolism (n = 6), coagulation (n = 4), complement inhibitors (n = 2), hormones (n = 9), direct attenuation of inflammation (n = 6), endothelial barrier modulators (n = 2), vasoactive agents (n = 19), or other agents (n = 6) involved in homeostasis. A total of 62 drugs were tested, of which seven were tested in multiple studies. An overview of all drugs and the dosage and timing of administration is presented in Table S3. The results as organized by working mechanism are presented below. In Table S4‐S7, the data are presented in table format, together with details of each individual experimental protocol.

#### 3.5.1 Antioxidants (n = 7)

**Figure 2:**
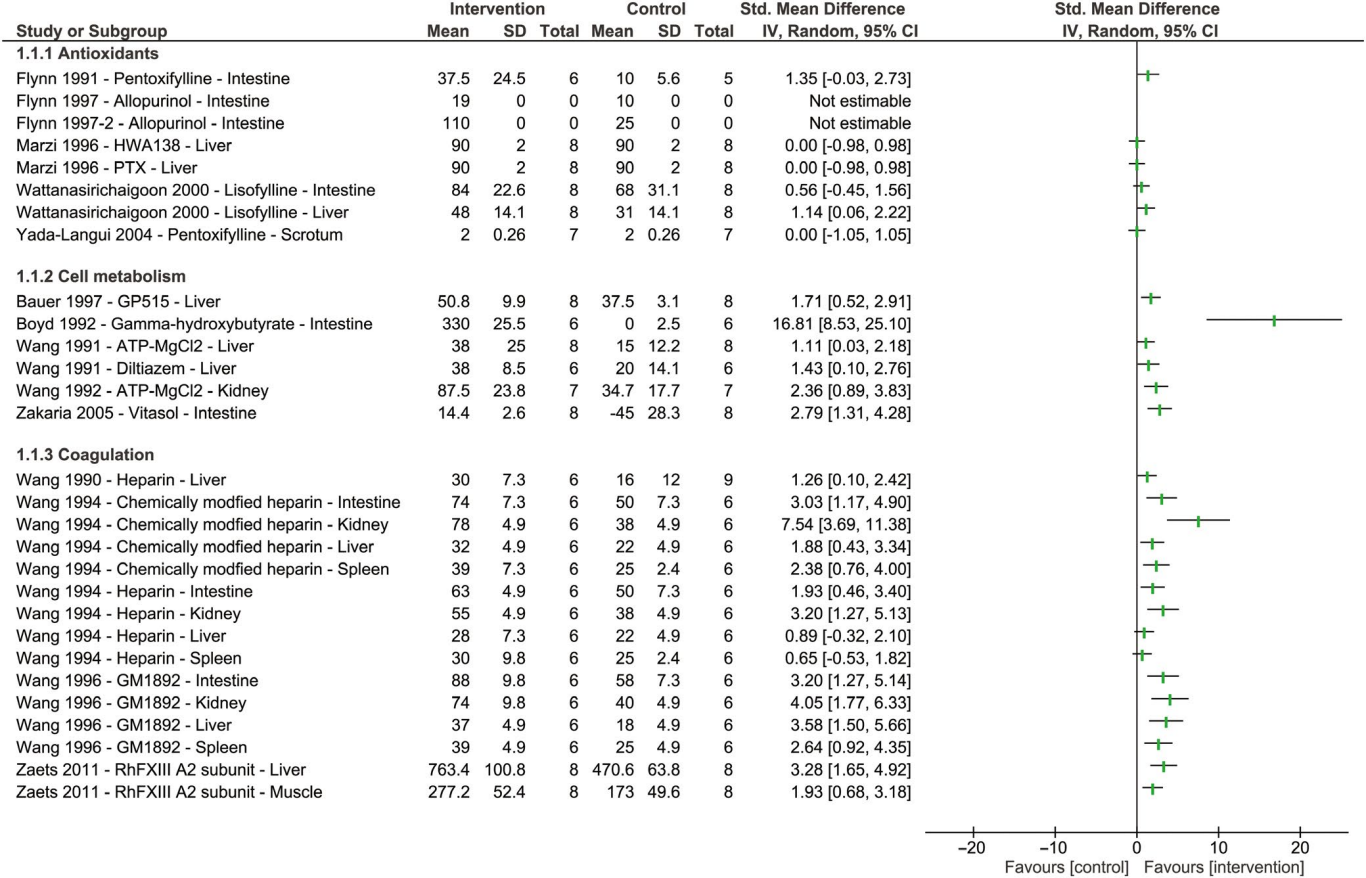
The effect of antioxidants and therapeutic agents targeting cell metabolism and coagulation on blood flow or red blood cell velocity following hemorrhagic shock and fluid resuscitation. Forest plots represent standardized mean differences accompanying 95% confidence intervals. Study names are reported as author, year of publication, name of therapeutic agent, and organ of measurement. Studies that did not report group sizes or standard deviations are shown as “not estimable.” No meta‐analysis was performed due to heterogeneity; therefore, no pooled effect is shown

Six studies tested the effect of antioxidants on blood flow,^23^, ^24^, ^25^, ^26^, ^27^, ^28^ and 50% reported an increase in blood flow compared to untreated controls (Figure 2, Table S4).^23^, ^24^, ^25^, ^27^ In total, seven antioxidants were tested. Blood flow was mainly measured in the intestinal or hepatic microvascular bed. The remaining studies reported no effect of the tested treatment.^26^, ^28^ Contrasting results were found by two different groups testing the effect of pentoxifylline,^25^, ^28^ as one group reported a restoration of intestinal blood flow following treatment,^25^ while the other group found no effect of pentoxifylline on RBC velocity in the scrotum.^28^ Two studies did not report the used group size and could therefore not be shown in the forest plot.^23^, ^24^

#### 3.5.2 Cell metabolism (n = 6)

Of the six studies targeting cell metabolism, five (83%) reported a restoration of blood flow following treatment with the drug (Figure 2, Table S4).^21^, ^29^, ^30^, ^31^, ^32^ Two of these studies tested the same drug, ATP‐MgCl_2_, on different organs, liver, and kidney, and reported a restoration of blood flow in both organs.^30^, ^32^ The remaining study reported a nonsignificant increase in hepatic blood flow following treatment with an adenosine kinase inhibitor.^33^ Boyd et al^29^ reported a complete stasis of blood flow in untreated controls, whereas in gamma‐hydroxybutyrate‐treated animals, intestinal blood flow was well maintained, leading to a large 95% confidence interval.

#### 3.5.3 Coagulation (n = 4)

Three studies tested the effect of heparin and chemically modified heparin on hepatic, intestinal, renal, and splenic blood flow (Figure 2, Table S4).^34^, ^35^, ^36^ Overall, they reported that the chemically modified variant of heparin was primarily effective in restoring blood flow following hemorrhagic shock and fluid resuscitation. The fourth study reported a restoration in blood flow after treatment with recombinant human FXIII, measured in both hepatic and muscular microcirculation.^37^

#### 3.5.4 Complement system (n = 2)

**Figure 3:**
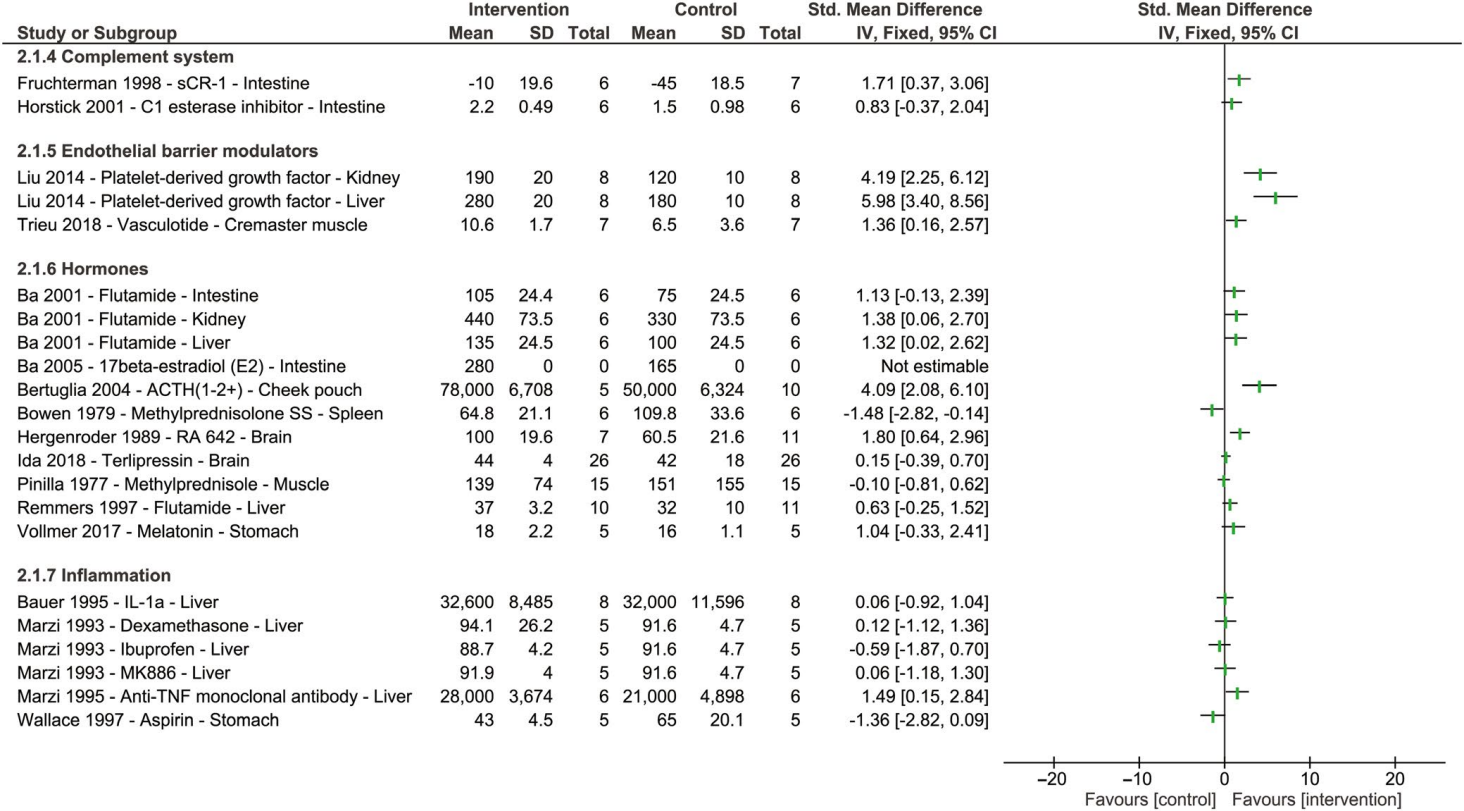
The effect of therapeutics targeting the complement system or systemic inflammation, endothelial barrier modulators, and hormones on blood flow or red blood cell velocity following hemorrhagic shock and fluid resuscitation. Forest plots represent standardized mean differences accompanying 95% confidence intervals. Study names are reported as author, year of publication, name of therapeutic agent, and organ of measurement. Studies that did not report group sizes or standard deviations are shown as “not estimable.” No meta‐analysis was performed due to heterogeneity; therefore, no pooled effect is shown

Two studies investigated targeting the complement system to restore blood flow and showed promising effects (Figure 3, Table S5).^38^, ^39^ Administration of recombinant human soluble complement receptor‐1 restored intestinal blood flow,^38^ and administration of a C1‐esterase inhibitor restored RBC velocity, but only slightly.^39^

#### 3.5.5 Endothelial barrier function (n = 2)

Both studies targeting the endothelial barrier with either the angiopoietin‐1 mimetic vasculotide or platelet‐derived growth factor showed a restoration in blood flow and/or perfusion following administration (Figure 3, Table S5).^40^, ^41^

#### 3.5.6 Hormones (n = 9)

Nine different hormones were reported, of which four (44%) restored blood flow following hemorrhagic shock and fluid resuscitation (Figure 3, Table S5).^42^, ^43^, ^44^, ^45^ Three of these effective treatment strategies targeted sex hormones with the use of testosterone receptor blocker flutamide^43^, ^45^ or by addition of 17B‐estradiol.^42^ The remaining studies either reported no effect (44%)^13^, ^19^, ^46^, ^47^ or a decrease in blood flow (11%) following treatment with hormones.^48^ One study did not report the used group size and could therefore not be shown in the forest plot.^42^

#### 3.5.7 Inflammation (n = 6)

Four studies tested the effect of six agents attenuating inflammation (Figure 3, Table S5). None of the therapeutic agents restored blood flow following hemorrhagic shock and fluid resuscitation.^22^, ^49^, ^50^, ^51^

#### 3.5.8 Vasoactive agents (n = 19)

**Figure 4:**
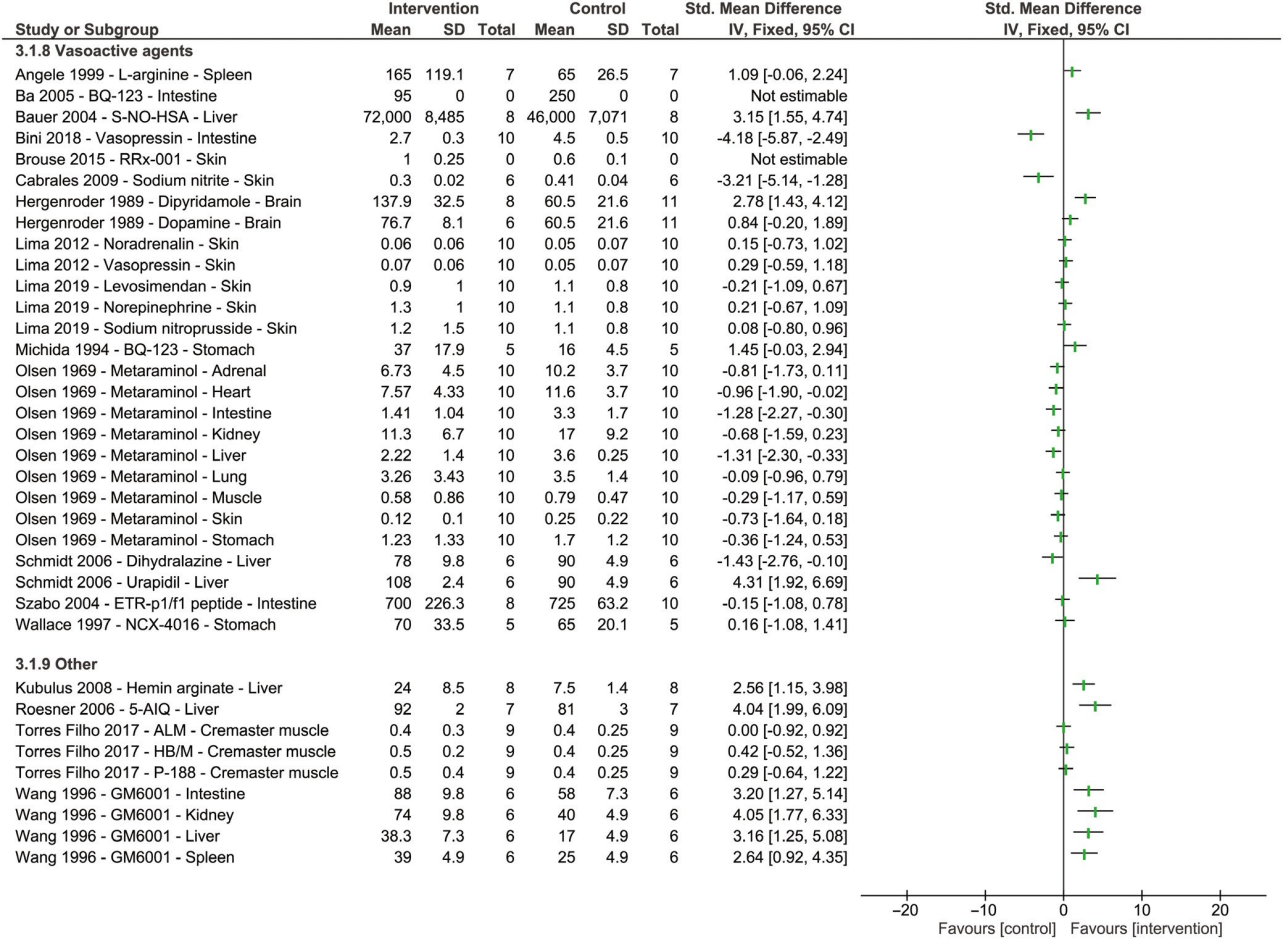
The effect of vasoactive agents or other therapeutics targeting homeostasis on blood flow or red blood cell velocity following hemorrhagic shock and fluid resuscitation. Forest plots represent standardized mean differences accompanying 95% confidence intervals. Study names are reported as author, year of publication, name of therapeutic agent, and organ of measurement. Studies that did not report group sizes or standard deviations are shown as “not estimable.” No meta‐analysis was performed due to heterogeneity; therefore, no pooled effect is shown. For Olsen et al,^20^ only results of the most clinically relevant model are shown, namely the conscious model

A total of 19 vasoactive agents were tested, of which six (32%) restored blood flow following hemorrhagic shock and fluid resuscitation (Figure 4, Table S6).^19^, ^42^, ^52^, ^53^, ^54^, ^55^ Eleven vasoactive agents (58%) did not affect blood flow.^19^, ^22^, ^56^, ^57^, ^58^, ^59^, ^60^ The remaining two studies (10%) reported a decrease in blood flow,^61^ or results varied markedly between organs.^20^ Vasopressin showed contrasting results with a decrease in intestinal blood flow,^61^ but no effect on dorsal blood flow.^57^ This may be because measurements were performed in different organs and different concentrations were given, and high concentrations of vasopressin are known to have adverse effects on microcirculatory perfusion. One study reported the effect of vasoactive agent metaraminol on blood flow under conscious and unconscious conditions in nine different organs.^20^ However, the reported results varied markedly and the experimental protocol is largely unclear which led to a high risk of bias. For this study, only the results of the clinically most relevant model, namely the conscious model, were shown in the forest plot. Two studies (10%) did not report the used group size and could therefore not be shown in the forest plot.^42^, ^54^

#### 3.5.9 Others (n = 6)

The remaining studies tested the effect of several other non‐fluid‐based therapeutic interventions involved in homeostasis (Figure 4, Table S7). Three of these studies reported a restoration in blood flow following treatment.^62^, ^63^, ^64^ These effective non‐fluid‐based treatment strategies consisted of hemin arginate, a compound inducing heme oxygenase 1,^62^ a PARP inhibitor,^63^ and GM6001, a matrix metalloproteinase inhibitor.^64^ The remaining three non‐fluid‐based therapeutic agents were not capable of restoring blood flow following hemorrhagic shock and fluid resuscitation.^65^ These compounds consisted of a combination of ALM, HB/M and poloxamer‐188 (P‐188). These therapeutics were tested based on previously reported promising results; however, their working mechanism remains unclear.

## 4 DISCUSSION

This systematic review demonstrates that non‐fluid‐based therapeutic interventions targeting in particular mitochondrial dysfunction, complement activation, and direct modulators of the endothelial barrier were able to restore microcirculatory perfusion following experimental hemorrhagic shock, in addition to fluid resuscitation. Non‐fluid‐based therapeutic treatments consisting of vasoactive agents, steroid hormones, or attenuation of systemic inflammation were less frequently able to restore microcirculatory perfusion. The evidence for these non‐fluid‐based therapeutic interventions comes from 48 preclinical studies and was mainly quantified in the hepatic and intestinal microvascular bed. Future studies should focus on confirming these mechanisms as target and eventually test these drugs in the clinical setting.

### 4.1 Complement system

The complement system is activated immediately following traumatic injury and continues following fluid resuscitation,^66^ and contributes to trauma‐related and ischemic tissue damage.^66^, ^67^ In this systematic search, two promising inhibitors of complement activation were described.^38^, ^39^ Inhibition via complement receptor‐1 restored mesenteric blood flow and endothelial cell function,^38^ while inhibition of the release of components C3a, C4a, and C5a via a C1‐esterase inhibitor reduced leukocyte adhesion which only slightly restored mesenteric blood flow.^39^ A recent review by Karasu et al summarized the current knowledge regarding targeting the complement system in critical illness. They reported that based on preclinical evidence, the complement system appears as an interesting targeting pathway.^68^ In the clinical setting, the use of a high‐dose C1‐esterase inhibitor improved survival rates for critically ill patients^69^ and although microcirculatory perfusion was not an outcome parameter, this confirms a potential promising use of complement inhibitors.

### 4.2 Coagulation

Coagulopathy following hemorrhagic shock and fluid resuscitation can lead to endothelial dysfunction.^6^, ^70^ Non‐fluid‐based therapeutic treatment targeting coagulation via fibrin stabilizing factor FXIII was effective in restoring hepatic microvascular blood flow and reducing pulmonary edema formation.^37^ However, targeting coagulopathy in critically ill remains debatable. Despite effectiveness of this treatment strategy, treatment of patients with a high risk of bleeding using anticoagulant therapeutics seems counterintuitive.^71^

The majority of experimental studies performed before 2000 were executed in pre‐heparinized animals, leaving the question unresolved whether heparin itself may be beneficial in restoring microcirculatory perfusion following hemorrhagic shock and fluid resuscitation.^34^ Although this was confirmed by Wang et al,^34^ the anticoagulant properties of heparin exclude its use in the clinical setting of hemorrhage and trauma. In this context, the same group investigated the effect of chemically modified heparin with reduced anticoagulant properties.^35^, ^36^ Treatment with this compound also restored renal, splenic, and intestinal microvascular blood flow and is proposed to function via inhibition of complement activation.^72^ Although both heparin and FXIII were effective in restoring microcirculatory perfusion, the usage of a treatment without anticoagulant properties, such as chemically modified heparin, is preferred when treating patients with an increased risk of bleeding. Chemically modified heparin is proposed to work via complement inhibition. Therefore, the use of complement inhibitors may be favored over heparin treatment strategy to restore microcirculatory perfusion following hemorrhagic shock and fluid resuscitation.

### 4.3 Mitochondrial function

Mitochondrial dysfunction following hemorrhagic shock and fluid resuscitation is characterized by increased ROS formation^73^ and decreased ATP production,^12^, ^74^ partly due to increased calcium content during ischemia.^75^ Several studies focused on improving ATP content,^21^, ^30^, ^31^, ^32^, ^33^ or reducing calcium levels with the use of a calcium antagonist,^21^ and all restored microcirculatory blood flow while using comparable models of moderate hemorrhagic shock. In addition, reducing ROS formation with a xanthine oxidase inhibitor, an antioxidant, restored mesenteric blood flow following hemorrhagic shock and fluid resuscitation.^23^, ^24^ Pentoxifylline, a methylxanthine derivate, was investigated in two different studies. One study reported an increase in blood flow following treatment,^25^ whereas the other study reported no effect on blood flow.^28^ Interestingly, the study that reported no effect of pentoxifylline used a more severe model of hemorrhagic shock compared to the study that reported an increase in blood flow, suggesting that the effectiveness of pentoxifylline is affected by the severity of hemorrhagic shock. In patients with septic shock, reducing calcium levels proved to be effective in restoring microcirculatory perfusion,^76^ confirming its clinical relevance as possible treatment strategy.

### 4.4 Endothelial barrier modulators

Improving endothelial barrier function appeared as a promising target to restore blood flow following hemorrhagic shock and fluid resuscitation.^40^, ^41^ Although no clinical studies have targeted endothelial barrier function yet, markers for endothelial cell activation and injury were upregulated in critically ill patients^77^, ^78^ and associated with multiple organ failure and unfavorable outcome.^77^ Consequently, restoring endothelial barrier function may be beneficial in improving microvascular blood flow following hemorrhagic shock and fluid resuscitation.^79^

### 4.5 Vasoactive agents

During hemorrhagic shock, the production of NO via eNOS^38^ is reduced due to diminished vascular endothelial shear stress and hypoxia,^80^ leading to vasoconstriction. As described in this review, restoration of NO levels via direct NO supplementation or administration of NO precursors restored blood flow,^52^, ^53^, ^54^ which even lasted up to 24 hours as reported by Bauer et al^53^ However, increasing NO levels via the NOS‐independent pathway by administration of nitrite only restored blood flow temporarily,^56^ suggesting that NO supplementation via eNOS is favorable. Other non‐fluid‐based agents with vasoactive properties showed conflicting results. Neither vasopressors^20^, ^57^, ^60^, ^61^ nor vasodilators^42^, ^55^, ^59^, ^60^ were effective in the preclinical setting. One of the studies investigating the effect of vasopressin reported a decrease in blood flow and an increase in capillary leak as a result of the given treatment,^61^ while another study reported no effect of vasopressin on blood flow.^57^ The use of vasopressin in the emergency setting remains under debate, as it has been associated with increased mortality in hemorrhagic shock patients.^81^ Overall, clinical trials targeting microcirculatory perfusion in critically ill patients remain inconclusive with regard to the use of vasoactive agents.^82^ Using vasoactive drugs seems mainly effective as hemodynamic support, however, exerts a range of unwarranted effects.^83^

### 4.6 Inflammation

The inflammatory response is characterized by the release of proinflammatory cytokines such as interleukin‐1β and TNF‐α and leukocyte adhesion.^51^, ^84^, ^85^ Interestingly, attenuation of these cytokines did not restore hepatic blood flow following experimental hemorrhagic shock and fluid resuscitation.^49^, ^51^ In parallel, direct attenuation of inflammation with dexamethasone, ibuprofen, a 5‐lipoxygenase inhibitor, or aspirin did not affect microcirculatory blood flow^22^, ^50^ in animals. The experimental models used in these studies were relatively homogenous as the majority used a moderate hemorrhagic shock model with a target blood pressure of 40 mm Hg and shock duration of one hour. Collectively, direct attenuation of inflammation does not appear to be effective in restoring microcirculatory perfusion following experimental hemorrhagic shock and fluid resuscitation. Similar results were reported by several clinical trials in patients with sepsis and septic shock, where treatment with anti‐inflammatory agents did not improve outcome of these patients.^86^ Accordingly, current trials focus on stimulating the immune response rather than inhibiting specific components of the immune system.^86^ However, future studies should elaborate on the efficiency of this approach.

### 4.7 Hormones

Gender differences have been a subject of interest for the past decade, revealing that females show improved microcirculatory function and reduced inflammatory response compared to males following trauma and hemorrhage.^42^, ^87^, ^88^ All hormonal treatments including either blocking the testosterone receptor^43^, ^45^ or the addition of estradiol steroid hormone 17B‐estradiol^42^ restored blood flow following experimental hemorrhagic shock and fluid resuscitation. However, conflicting results were found when administrating steroid hormones in animals.^44^, ^46^, ^48^ One study even reported a decrease in splenic blood flow following treatment with a steroid hormone.^48^ Important to note, however, is the rigorous hemorrhagic shock model used in this study, as the animals were kept in shock for 3.5 hours, which limits the translation of this model to the clinical setting. The use of steroid hormones as treatment strategy to restore blood flow is based on the possible attenuation of systemic inflammation. The lack of the capability to restore blood flow is therefore in line with previously discussed anti‐inflammatory agents. Comparable results were reported by Martino et al,^89^ where the authors discussed conflicting results of corticosteroids on survival, tested in major clinical trials with critically ill patients.

Collectively, improving microcirculatory blood flow following hemorrhagic shock and fluid resuscitation by blocking or stimulating sex hormones appears effective. A clinical trial showing a lower in‐hospital mortality rate in female patients confirms the importance of investigating gender differences in severely injured patients.^90^ As also shown in the current review, the majority of the therapeutic agents is tested in male animals, limiting translation to the female population due to hormonal differences. Further research is therefore not only necessary to confirm the efficiency of these therapeutics in females, but should also elaborate on the role of sex hormones as treatment strategy.

## 5 LIMITATIONS

The quality and translational value of the included studies varied markedly. As only 24% of the studies reported blinding of the investigators or outcome assessors at any point, a cautious approach to their interpretation is necessary. Most of the studies were performed in rodents rather than in large animals, and only a few studies reported a power calculation. Moreover, most of the microcirculatory perfusion measurements were performed in either the liver or the intestinal bed. Although more difficult to access, one would prefer to measure the effect of therapeutic agents on pulmonary and renal function in order to increase translatability,^6^ as these organs are particularly susceptible to microvascular failure in the earliest phase following hemorrhagic shock.^91^ Nonetheless, as the splanchnic circulation is particularly susceptible to hemorrhagic shock and fluid resuscitation, these results are of additional value. Due to the heterogeneity of studies, meta‐analysis was unwarranted, limiting the ability to assess the relative effect of a specific targeting pathway. Therefore, this study was unable to identify the most promising treatment strategy. Nevertheless, this systematic review provides an overview of published literature on non‐fluid‐based therapeutic treatment interventions to restore hemorrhagic shock and fluid resuscitation‐related microcirculatory perfusion deficits in animals. It should, however, be noted that the current review summarizes preclinical evidence. Translation of the current results into the clinical setting, with ultimately improvement of patient outcome, still takes several steps. At this moment, clinical evidence to strengthen the evidence of potential treatment strategies is rare, due to the complex origin of current patient population.

## 6 PERSPECTIVES

Microcirculatory perfusion disturbances following hemorrhagic shock and fluid resuscitation are associated with multiple organ failure and increased mortality. As standard fluid resuscitation only restores macrohemodynamics, additional treatment with a non‐fluid‐based therapeutic agent is essential to restore microcirculatory perfusion. As reported in the current review, targeting mitochondrial function, complement activation, or endothelial barrier function, the majority of the tested agents contributed to a restoration of microcirculatory perfusion following hemorrhagic shock. We emphasize that these therapeutic agents were given in addition to standard fluid resuscitation and do not replace a transfusion protocol. Future studies should reveal whether these therapeutics are also effective in restoring microcirculatory perfusion in the clinical setting. As gender differences play a role in the microcirculatory and inflammatory response to hemorrhagic shock, further research is necessary to clarify the effect of treatment interventions focusing on sex hormones.

## Supporting information

Supinfo
