# Supplementary material for: Therapeutic interventions to restore microcirculatory perfusion following experimental hemorrhagic shock and fluid resuscitation: A systematic review
Source: Microcirculation. 2020 Aug 20;27(8):e12650. doi: 10.1111/micc.12650 (PMC7757213; doi:10.1111/micc.12650)

**Therapeutic interventions to restore microcirculatory perfusion following experimental hemorrhagic shock and fluid resuscitation: a systematic review**

**Supplemental digital content**

Anoek L.I. van Leeuwen^1,2^, BSc, [a.vanleeuwen1@amsterdamumc.nl](mailto:a.vanleeuwen1@amsterdamumc.nl)

Nicole A.M. Dekker^1,2^, MD, [n.dekker@amsterdamumc.nl](mailto:n.dekker@amsterdamumc.nl)

Elise P. Jansma^3,4^, MSc, [i.jansma@amsterdamumc.nl](mailto:i.jansma@amsterdamumc.nl)

Christa Boer^1^, PhD, [c.boer@amsterdamumc.nl](mailto:c.boer@amsterdamumc.nl)

Charissa E. van den Brom^1,2, +^, PhD, [c.vandenbrom@amsterdamumc.nl](mailto:c.vandenbrom@amsterdamumc.nl)

^1^Department of Anesthesiology, Experimental Laboratory for VItal Signs, Amsterdam UMC, Vrije Universiteit, Amsterdam Cardiovascular Sciences, Amsterdam, The Netherlands;

^2^Department of Physiology, Amsterdam UMC, Vrije Universiteit, Amsterdam Cardiovascular Sciences, Amsterdam, The Netherlands;

^3^Department of Epidemiology and Biostatistics, Amsterdam UMC, Vrije Universiteit, Amsterdam Public Health research institute, Amsterdam, The Netherlands.

^4^Medical Library, Vrije Universiteit, Amsterdam, The Netherlands.

**Appendix S1: Supplemental methods**

**Search terms for Pubmed**

**#1 Hemorrhagic Shock**

"Shock, Hemorrhagic"[Mesh] OR hemorrhagic shock[tiab] OR haemorrhagic shock[tiab] OR hemorrhage shock[tiab] OR haemorrhage shock[tiab] OR bleeding shock[tiab]

**#2 Capillaries**

"microvessels"[Mesh] OR "Endothelium"[Mesh:NoExp] OR "Tunica Intima"[Mesh] OR endothel*[tiab] OR intima[tiab] OR capill*[tiab] OR microvasc* OR micro-vasc*[tiab] OR microvess*[tiab] OR micro-vess*[tiab] OR micro blood vessel*[tiab] OR small blood vessel*[tiab] OR vas capillare[tiab]

**#3 Perfusion & flow (primary outcome)**

"Perfusion"[Mesh] OR "Blood Flow Velocity"[Mesh] OR Perfusion*[tiab] OR reperfusion[tiab] OR hypoperfusion[tiab] OR microperfusion[tiab] OR flow[tiab] OR bloodflow[tiab] OR blood stream[tiab]

**Search terms for Embase**

**#1 Hemorrhagic Shock**

'hemorrhagic shock'/exp OR 'hemorrhagic shock':ti,ab OR 'haemorrhagic shock':ti,ab OR 'hemorrhage shock':ti,ab OR 'haemorrhage shock':ti,ab OR 'bleeding shock':ti,ab

**#2 Capillaries**

'microvasculature'/exp OR 'endothelium'/de OR 'capillary endothelium'/exp OR 'intima'/exp OR 'vascular endothelium'/exp OR endothel*:ti,ab OR intima:ti,ab OR capill*:ti,ab OR microvasc* OR 'micro-vasc*':ti,ab OR microvess*:ti,ab OR 'micro-vess*':ti,ab OR 'micro blood vessel*':ti,ab OR 'small blood vessel*':ti,ab OR 'vas capillare':ti,ab

**#3 Perfusion & flow (primary outcome)**

'perfusion'/exp OR 'blood flow'/exp OR Perfusion:ti,ab OR reperfusion:ti,ab OR hypoperfusion:ti,ab OR microperfusion:ti,ab OR flow:ti,ab OR bloodflow:ti,ab OR 'blood stream':ti,ab

**Search terms for Cochrane Library/CENTRAL**

**#1 Hemorrhagic Shock**

"hemorrhagic shock" OR "haemorrhagic shock" OR "hemorrhage shock" OR "haemorrhage shock" OR "bleeding shock"

**#2 Capillaries**

endothel* OR intima OR capill* OR microvasc* OR micro-vasc* OR microvess* OR micro-vess* OR micro blood vessel* OR small blood vessel* OR "vas capillare"

**#3 Perfusion & flow**

Perfusion* OR reperfusion OR hypoperfusion OR microperfusion OR flow OR bloodflow OR blood stream

**Table S1.** PRISMA checklist

| **Section/topic** | **#** | **Checklist item** | **Reported on page #** |
| --- | --- | --- | --- |
| **TITLE** | | |  |
| Title | 1 | Identify the report as a systematic review, meta-analysis, or both. | 1 |
| **ABSTRACT** | | |  |
| Structured summary | 2 | Provide a structured summary including, as applicable: background; objectives; data sources; study eligibility criteria, participants, and interventions; study appraisal and synthesis methods; results; limitations; conclusions and implications of key findings; systematic review registration number. | 2 |
| **INTRODUCTION** | | |  |
| Rationale | 3 | Describe the rationale for the review in the context of what is already known. | 4-5 |
| Objectives | 4 | Provide an explicit statement of questions being addressed with reference to participants, interventions, comparisons, outcomes, and study design (PICOS). | 4-5 |
| **METHODS** | | |  |
| Protocol and registration | 5 | Indicate if a review protocol exists, if and where it can be accessed (e.g., Web address), and, if available, provide registration information including registration number. | 6 |
| Eligibility criteria | 6 | Specify study characteristics (e.g., PICOS, length of follow-up) and report characteristics (e.g., years considered, language, publication status) used as criteria for eligibility, giving rationale. | 6 |
| Information sources | 7 | Describe all information sources (e.g., databases with dates of coverage, contact with study authors to identify additional studies) in the search and date last searched. | 6-7 |
| Search | 8 | Present full electronic search strategy for at least one database, including any limits used, such that it could be repeated. | Suppl. methods |
| Study selection | 9 | State the process for selecting studies (i.e., screening, eligibility, included in systematic review, and, if applicable, included in the meta-analysis). | 7 |
| Data collection process | 10 | Describe method of data extraction from reports (e.g., piloted forms, independently, in duplicate) and any processes for obtaining and confirming data from investigators. | 7 |
| Data items | 11 | List and define all variables for which data were sought (e.g., PICOS, funding sources) and any assumptions and simplifications made. | 7 |
| Risk of bias in individual studies | 12 | Describe methods used for assessing risk of bias of individual studies (including specification of whether this was done at the study or outcome level), and how this information is to be used in any data synthesis. | 8 |
| Summary measures | 13 | State the principal summary measures (e.g., risk ratio, difference in means). | 8 |
| Synthesis of results | 14 | Describe the methods of handling data and combining results of studies, if done, including measures of consistency (e.g., I^2^) for each meta-analysis. | 8 |

Page 1 of 2

| **Section/topic** | **#** | **Checklist item** | **Reported on page #** |
| --- | --- | --- | --- |
| Risk of bias across studies | 15 | Specify any assessment of risk of bias that may affect the cumulative evidence (e.g., publication bias, selective reporting within studies). | 8 |
| Additional analyses | 16 | Describe methods of additional analyses (e.g., sensitivity or subgroup analyses, meta-regression), if done, indicating which were pre-specified. | 8 |
| **RESULTS** | | |  |
| Study selection | 17 | Give numbers of studies screened, assessed for eligibility, and included in the review, with reasons for exclusions at each stage, ideally with a flow diagram. | p. 9 +  Fig.1 |
| Study characteristics | 18 | For each study, present characteristics for which data were extracted (e.g., study size, PICOS, follow-up period) and provide the citations. | p. 9 +  Table 1 |
| Risk of bias within studies | 19 | Present data on risk of bias of each study and, if available, any outcome level assessment (see item 12). | p. 10 +  Suppl table 2 |
| Results of individual studies | 20 | For all outcomes considered (benefits or harms), present, for each study: (a) simple summary data for each intervention group (b) effect estimates and confidence intervals, ideally with a forest plot. | p. 10-12  Fig 2-4 |
| Synthesis of results | 21 | Present results of each meta-analysis done, including confidence intervals and measures of consistency. | N/A |
| Risk of bias across studies | 22 | Present results of any assessment of risk of bias across studies (see Item 15). | Suppl fig 1 |
| Additional analysis | 23 | Give results of additional analyses, if done (e.g., sensitivity or subgroup analyses, meta-regression [see Item 16]). | N/A |
| **DISCUSSION** | | |  |
| Summary of evidence | 24 | Summarize the main findings including the strength of evidence for each main outcome; consider their relevance to key groups (e.g., healthcare providers, users, and policy makers). | 13-17 |
| Limitations | 25 | Discuss limitations at study and outcome level (e.g., risk of bias), and at review-level (e.g., incomplete retrieval of identified research, reporting bias). | 16-17 |
| Conclusions | 26 | Provide a general interpretation of the results in the context of other evidence, and implications for future research. | 17 |
| **FUNDING** | | |  |
| Funding | 27 | Describe sources of funding for the systematic review and other support (e.g., supply of data); role of funders for the systematic review. | 1 |

*From:*  Moher D, Liberati A, Tetzlaff J, Altman DG, The PRISMA Group (2009). Preferred Reporting Items for Systematic Reviews and Meta-Analyses: The PRISMA Statement. PLoS Med 6(7): e1000097. doi:10.1371/journal.pmed1000097

For more information, visit: **www.prisma-statement.org**.

Page 2 of 2

**Table S2.** SYRCLE’s risk of bias tool

|  |  | 1. Randomization group allocation? | 2. Randomization treatment administration? | 3. Random housing? | 4. Random selection outcome? | 5. Care givers / investigators blinded? | 6. Outcome assessor blinded? | 7. Groups similar at baseline? | 8. Incomplete outcome data addressed? | 9. Free of selective outcome reporting? | 10. Other risks of bias? | 11. Drug tested in sham group? | 12. Power calculation? | Randomization at any point? | Blinding at any point? |
| --- | --- | --- | --- | --- | --- | --- | --- | --- | --- | --- | --- | --- | --- | --- | --- |
| Angele | 1999 | Yes | Yes | Unclear | Unclear | No | No | Unclear | Unclear | No | Yes | Yes | No | **yes** | **no** |
| Ba | 2001 | No | No | Unclear | Unclear | No | No | Unclear | Unclear | No | Yes | No | No | **no** | **no** |
| Ba | 2005 | No | No | Unclear | Unclear | No | No | Unclear | Unclear | No | Yes | Yes | No | **no** | **no** |
| Bauer | 1995 | Yes | Yes | Unclear | Unclear | Yes | Yes | Yes | Unclear | Yes | Yes | No | No | **yes** | **yes** |
| Bauer | 1997 | Yes | Yes | Unclear | Unclear | Yes | Yes | Yes | Unclear | Yes | Yes | Yes | No | **yes** | **yes** |
| Bauer | 2004 | Yes | Yes | Unclear | Unclear | No | Yes | Yes | Unclear | Yes | Yes | Yes | No | **yes** | **yes** |
| Bertuglia | 2004 | Yes | Yes | Unclear | Unclear | No | No | Yes | Unclear | Yes | Yes | No | No | **yes** | **no** |
| Bini | 2018 | Yes | Yes | Unclear | Unclear | No | No | Yes | Yes | Yes | Yes | No | No | **yes** | **no** |
| Bowen | 1979 | No | No | Unclear | Unclear | No | No | Yes | Unclear | Yes | Yes | No | No | **no** | **no** |
| Boyd | 1992 | No | No | Unclear | Unclear | No | No | Yes | Unclear | Yes | Yes | No | No | **no** | **no** |
| Brouse | 2015 | Yes | Yes | Unclear | Unclear | No | No | Yes | Unclear | Yes | No | No | No | **yes** | **no** |
| Cabrales | 2009 | Yes | Yes | Unclear | No | No | No | Unclear | Yes | Yes | Yes | No | No | **yes** | **no** |
| Flynn | 1991 | No | Yes | Unclear | No | Yes | No | Yes | Unclear | Yes | Yes | No | No | **yes** | **yes** |
| Flynn | 1997 | No | Yes | Unclear | No | No | No | No | Yes | Yes | Yes | No | No | **yes** | **no** |
| Flynn | 1997 | No | Yes | Unclear | No | No | No | No | Yes | Yes | Yes | No | No | **yes** | **no** |
| Fruchterman | 1998 | No | No | Unclear | Unclear | No | No | Yes | Unclear | No | Yes | Yes | No | **no** | **no** |
| Hergenroder | 1989 | No | No | Unclear | Unclear | No | No | Yes | Unclear | Yes | Yes | No | No | **no** | **no** |
| Ida | 2001 | Yes | Yes | Unclear | Unclear | No | No | Unclear | Yes | Yes | Yes | No | Yes | **yes** | **no** |
| Horstick | 2018 | Yes | Yes | Unclear | Unclear | No | Yes | Yes | Yes | Yes | Yes | No | No | **yes** | **yes** |
| Kubulus | 2008 | No | No | Unclear | Unclear | No | Yes | Yes | Unclear | Yes | Yes | No | No | **no** | **yes** |
| Lima | 2012 | Yes | Yes | Unclear | Unclear | No | No | Unclear | Yes | Yes | Yes | No | No | **yes** | **no** |
| Lima | 2019 | No | No | Unclear | Unclear | No | No | Yes | Unclear | Yes | Yes | No | No | **no** | **no** |
| Liu | 2014 | Yes | Yes | Unclear | Unclear | No | No | Yes | Yes | Yes | Yes | No | Yes | **yes** | **no** |
| Marzi | 1993 | Yes | Yes | Unclear | Unclear | No | No | Unclear | Unclear | No | No | No | No | **yes** | **no** |
| Marzi | 1995 | No | No | Unclear | Unclear | No | No | Yes | Unclear | Yes | Yes | No | No | **no** | **no** |
| Marzi | 1996 | Yes | Yes | Unclear | Unclear | Yes | Yes | Yes | Unclear | Yes | Yes | No | No | **yes** | **yes** |
| Michida | 1994 | No | No | Unclear | Unclear | No | No | Unclear | Unclear | No | Yes | No | No | **no** | **no** |
| Olsen | 1969 | No | No | Unclear | Unclear | No | No | Unclear | Unclear | Yes | Yes | No | No | **no** | **no** |
| Pinilla | 1977 | Yes | Yes | Unclear | Unclear | No | No | Yes | Yes | Yes | Yes | No | No | **yes** | **no** |
| Remmers | 1997 | No | No | Unclear | Unclear | No | No | Unclear | Unclear | Yes | Yes | Yes | No | **no** | **no** |
| Roesner | 2006 | Yes | Yes | Unclear | Unclear | No | No | Yes | Unclear | No | Yes | Yes | No | **yes** | **no** |
| Schmidt | 2006 | Yes | Yes | Unclear | Unclear | No | No | Unclear | Unclear | Yes | No | No | No | **yes** | **no** |
| Szabo | 2004 | Yes | Yes | Unclear | Unclear | No | No | Yes | Unclear | Yes | Yes | No | No | **yes** | **no** |
| Torres Filho | 2017 | Yes | Yes | Unclear | Unclear | Yes | Yes | Unclear | Unclear | Yes | Yes | No | No | **yes** | **yes** |
| Trieu | 2018 | Yes | Yes | Unclear | Unclear | No | Yes | Yes | Yes | Yes | No | Yes | Yes | **yes** | **yes** |
| Vollmer | 2017 | Yes | Yes | Unclear | Unclear | Yes | No | No | Unclear | Yes | Yes | Yes | Yes | **yes** | **yes** |
| Wallace | 1997 | No | No | Unclear | Unclear | No | No | Unclear | Unclear | No | No | No | No | **no** | **no** |
| Wang | 1990 | No | No | Unclear | Unclear | No | No | Unclear | Unclear | Yes | Yes | Unclear | No | **no** | **no** |
| Wang | 1991 | No | No | Unclear | Unclear | No | No | Unclear | Unclear | Yes | Yes | Yes | No | **no** | **no** |
| Wang | 1991 | No | No | Unclear | Unclear | No | No | Unclear | Yes | Yes | Yes | Yes | No | **no** | **no** |
| Wang | 1992 | No | No | Unclear | Unclear | No | No | Unclear | Yes | Yes | Yes | No | No | **no** | **no** |
| Wang | 1994 | No | No | Unclear | Unclear | No | No | Unclear | Yes | Yes | Yes | No | No | **no** | **no** |
| Wang | 1996 | No | No | Unclear | Unclear | No | No | Unclear | Yes | Yes | Yes | No | No | **no** | **no** |
| Wang | 1996 | No | No | Unclear | Unclear | No | No | Unclear | Yes | Yes | Yes | No | No | **no** | **no** |
| Wattanasirichaigoon | 2000 | No | Yes | Unclear | Unclear | Yes | No | Yes | Unclear | No | Yes | No | No | **yes** | **yes** |
| Yada-Langui | 2004 | Yes | Yes | Unclear | Unclear | No | No | Unclear | Unclear | Yes | Yes | No | No | **yes** | **no** |
| Zaets | 2011 | Yes | Yes | Unclear | Unclear | Yes | No | Yes | Unclear | Yes | Yes | Yes | No | **yes** | **yes** |
| Zakaria | 2005 | Yes | Yes | Unclear | Unclear | No | No | Yes | Unclear | Yes | Yes | Yes | No | **yes** | **no** |

Quality assessment based on SYRCLE Risk of Bias tool. “Yes”, “Unclear”, “No” indicates “low”, “unclear”, and “high” risk of bias, respectively

**Table S3.** Details of therapeutic interventions and measurement techniques

|  |  |  | Treatment | | | | Outcome measurement | |
| --- | --- | --- | --- | --- | --- | --- | --- | --- |
|  |  |  | **Drug name** | **Control** | **Dose** | **Time of administration** | **Method** | **Endpoint** |
| Antioxidant | Flynn | 1991 | Pentoxifylline | Saline | 49 mg/kg | Post-shock | LDF | RBC velocity |
|  | Flynn | 1997 | Allopurinol | Saline | 50 mg/kg,  25 mg/kg/h | Post-shock,  post-resuscitation | IVM and LDF | Blood flow |
|  | Flynn | 1997 | Allopurinol | Saline | 50 mg/kg,  25 mg/kg/h | Post-shock,  post-resuscitation | IVM and LDF | Blood flow |
|  | Marzi | 1996 | PTX | RL | 25 mg/kg | Post-shock | IVM with FITC labeled RBCs | Blood flow,  RBC velocity |
|  | Marzi | 1996 | HWA138 | RL | 25 mg/kg | Post-shock | IVM with FITC labeled RBCs | Blood flow,  RBC velocity |
|  | Wattanasirichaigoon | 2000 | Lisofylline | RL | 52.5 mg/kg | Post-shock | LDF | Blood flow |
|  | Yada-Langui | 2004 | Pentoxifylline | None | 25 mg/kg | Post-shock | LDF | RBC velocity |
| Cell metabolism | Bauer | 1997 | GP515 | Saline | 0.25 mg/kg | Pre-shock | IVM | Blood flow |
|  | Boyd | 1992 | Gamma-hydroxybutyrate (GHB) | Saline | 600 mg/kg | Pre-shock | IVM with FITC-labeled RBCs | RBC velocity |
|  | Wang | 1991 | Diltiazem | Saline | 400 ug/kg | Post-resuscitation | LDF | Blood flow |
|  | Wang | 1991 | ATP-MgCl2 | Saline | 50 µmol/kg | Post-resuscitation | LDF | Blood flow |
|  | Wang | 1992 | ATP-MgCl2 | Saline | 50 umol/kg | Post-resuscitation | LDF | Blood flow |
|  | Zakaria | 2005 | Vitasol | Vehicle | 2.5 mmol/l | Post-resuscitation | IVM | Blood flow |
| Complement | Fruchterman | 1998 | Recombinant human soluble complement receptor-1 (sCR1) | Saline | 15 mg/kg | Post-shock | IVM and LDF | Blood flow |
|  | Horstick | 2018 | Terlipressin | None | 10 ug/100g | Post-shock | CFM | Area of perfused vessels |
| Coagulation | Wang | 1990 | Heparin | None | 2 U/g | Pre-shock | LDF | Blood flow |
|  | Wang | 1994 | Heparin | Saline | 7 mg/kg | Post-resuscitation | LDF | Blood flow |
|  | Wang | 1994 | Chemically modified heparin | Saline | 7 mg/kg | Post-resuscitation | LDF | Blood flow |
|  | Wang | 1996 | GM1892 | Saline | 7 mg/kg | Post-resuscitation | LDF | Blood flow |
|  | Zaets | 2011 | Recombinant human FXIII A2 subunit | Vehicle | 1 mg/kg | Post-shock | OxyFlo (LDF / digital signal processing) | Blood flow |
| Endothelial barrier modulator | Liu | 2014 | Platelet-derived growth factor | RL | 1, 3.5, 7 and 15 ug/kg | Post-shock | LDF | Blood flow |
|  | Trieu | 2018 | Vasculotide | PBS | 200 ng/rat | Post-shock | IVM | Perfusion |
| Hormones | Ba | 2001 | Flutamide | Propanediol | 25 mg/kg | Pre-shock | 85-labeled microspheres | Blood flow |
|  | Ba | 2005 | 17beta-estradiol (E2) | None | 0.015-0.03 mg/rat | Pre-shock | Perfusion pressure in isolated intestine | Perfusion flow |
|  | Bertuglia | 2004 | Adrenocorticotropic-stimulating  hormone (ACTH (1-2+)) | None | 160 ug/kg | Post-shock | IVM with FITC-dextrans | Blood flow, RBC velocity |
|  | Bowen | 1979 | Methylprednisolone sodium succinate | None | 30 mg/kg | Post-shock | Electromagnetic square wave flow amplifier | Blood flow |
|  | Hergenroder | 1989 | Dopamine | Saline | 0.1 mg/kg | Post-shock | LDF | Blood flow |
|  | Ida | 2001 | C1-INH | Saline | 100 IU/kg | Post-shock | IVM | RBC velocity |
|  | Pinilla | 1977 | Methylprednisolone | None | 30 mg/kg | Post-shock | Xenon-133 clearance | Blood flow |
|  | Remmers | 1997 | Flutamide | Propanediol | 25 mg/kg | Post-resuscitation | LDF | Blood flow |
|  | Vollmer | 2017 | Melatonin | Ethanol solution | 100 ug/kg | Pre-shock | LDF | Blood flow,  RBC velocity |
| Inflammation | Bauer | 1995 | IL-1ra | Saline | 15 mg/kg | Pre-shock and post-shock | IVM | Blood flow |
|  | Marzi | 1993 | Ibuprofen | Saline | 15mg/kg | Post-shock | IVM with fluorescent labeled leukocytes | Perfusion |
|  | Marzi | 1993 | MK886 | Saline | 10 mg/kg | Post-shock | IVM with fluorescent labeled leukocytes | Perfusion |
|  | Marzi | 1993 | Dexamethasone | Saline | 5 mg/kg | Post-shock | IVM with fluorescent labeled leukocytes | Perfusion |
|  | Marzi | 1995 | Anti-TNF monoclonal antibody (TN3) | Saline | 2 mg/kg | Pre-shock | IVM with fluorescent labeled leukocytes | Blood flow |
|  | Wallace | 1997 | Aspirin | None | 50 mg/kg | Pre-shock | LDF | Blood flow |
| Vasoactive agents | Angele | 1999 | L-arginine | Saline | 300 mg/kg | Post-shock | 85SR-labeled microspheres | Blood flow |
|  | Ba | 2005 | BQ-123 | None | 10 ng/ml | Pre-shock | Perfusion pressure in isolated intestine | Perfusion flow |
|  | Bauer | 2004 | S-nitroso-albumin (S-NO-HSA) | Vehicle | 10 umol/kg | Post-shock | IVM | Blood flow |
|  | Bini | 2018 | Vasopressin | None | 0.04 U/kg/min | Post-shock | Perivascular probe | Blood flow |
|  | Brouse | 2015 | RRx-001 | None | 2 mg/ml | Post-shock | IVM | Blood flow, FCD |
|  | Cabrales | 2009 | Sodium nitrite | Saline | 0, 10 and 50 uM | Post-shock | IVM | Blood flow, FCD |
|  | Hergenroder | 1989 | RA 642 | Saline | 2 mg/kg | Post-shock | LDF | Blood flow |
|  | Hergenroder | 1989 | Dipyridamole | Saline | 2 mg/kg | Post-shock | LDF | Blood flow |
|  | Lima | 2012 | Vasopressin | Saline | 0.006 UI/kg | Post-shock | IVM | RBC velocity, FCD |
|  | Lima | 2012 | Noradrenaline | Saline | 120 ug/kg | Post-shock | IVM | RBC velocity, FCD |
|  | Lima | 2019 | Sodium nitroprusside | RL | 0.03 μg/kg/min | Post-shock | IVM | RBC velocity, FCD |
|  | Lima | 2019 | Norepinephrine | RL | 2 μg/kg/min | Post-shock | IVM | RBC velocity, FCD |
|  | Lima | 2019 | Levosimendan | RL | 0.3 μg/kg/min | Post-shock | IVM | RBC velocity, FCD |
|  | Michida | 1994 | BQ-123 | PBS | 10 mg/ml | Post-shock | LDF | Blood flow |
|  | Olsen | 1969 | Metaraminol | None | ND | Post-shock | Radioactive potassium uptake | Blood flow |
|  | Schmidt | 2006 | Urapidil | Aqua ad injectabilia | 3 mg/kg | Post-shock | LDF | Blood flow |
|  | Schmidt | 2006 | Dihydralazine | Aqua ad injectabilia | 1.5 mg/kg | Post-shock | LDF | Blood flow |
|  | Szabo | 2004 | ETR-p1/f1 peptide | None | 100 nmol/kg | Post-shock | OPS | RBC velocity, FCD |
|  | Wang | 1996 | GM6001 | Saline | 100 mg/kg | Post-resuscitation | LDF | Blood flow |
| Others | Kubulus | 2008 | Hemin arginate | RL | 5 mg/kg | Pre-shock | IVM with FITC-labeled RBCs | Blood flow, RBC velocity |
|  | Roesner | 2006 | 5-AIQ | PBS | 3 mg/kg | Post-shock | IVM with sodium fluorescein | Perfusion rate |
|  | Torres Filho | 2017 | Adenosine-lidocaine-magnesium (ALM) | None | 3.5 mL/kg | Post-shock | IVM | Blood flow, RBC velocity |
|  | Torres Filho | 2017 | Beta-hydroxybutyrate + melatonin (HB/M) | None | HB: 4M + melatonin: 43 mM | Post-shock | IVM | Blood flow, RBC velocity |
|  | Torres Filho | 2017 | Poloxamer -188 | None | 200 mg/kg | Post-shock | IVM | Blood flow, RBC velocity |

Fluids: RL, Ringer’s lactate; PBS, phosphate buffered saline.

Techniques: OPS, orthogonal polarization spectral imaging; IVM, intravital microscopy; CFM, confocal microscopy; LDF, laser Doppler flowmetry; FITC, fluorescein isothiocyanate.

Endpoints: RBC velocity, red blood cell velocity; FCD, functional capillary density.

**Table S4.** Summary of study findings regarding antioxidants and therapeutic agents targeting cell metabolism and coagulation

|  | **First author** | **Year** | **Name of drug** | **Group size (n)** | **Depth of HS (mmHg)** | **Duration HS (min)** | **Measurement time point after resuscitation** | **Outcome** | **Organ** | **Summary** | |
| --- | --- | --- | --- | --- | --- | --- | --- | --- | --- | --- | --- |
| **ANTIOXIDANTS** | Flynn | 1991 | Pentoxifylline | 5-6 | 50 | 45 | 2h | Blood flow | Intestine | ↑ | |
|  | Flynn | 1997 | Allopurinol | ND | 65 | 45 | 2h | Blood flow | Intestine | ↑ | |
|  | Flynn | 1997 | Allopurinol | ND | 50 | 60 | 1.5h | Blood flow | Intestine | ↑ | |
|  | Marzi | 1996 | PTX | 8 | 40 | 60 | 2.5h | Blood flow | Liver | = | |
|  | Marzi | 1996 | HWA138 | 8 | 40 | 60 | 2.5h | Blood flow | Liver | = | |
|  | Wattanasirichaigoon | 2000 | Lisofylline | 8 | 30 | 90 | 1.5h | Blood flow | Intestine | = | |
|  |  |  |  |  |  |  |  |  | Liver | ↑ | |
|  | Yada-Langui | 2004 | Pentoxifylline | 7 | 35 | 60 | 2h | RBC velocity | Scrotum | = | |
| **CELL METABOLISM** | Bauer | 1997 | GP515 | 8 | 40 | 60 | 5h | Blood flow | Liver | = |  |
|  | Boyd | 1992 | gamma-hydroxybutyrate (GHB) | 6 | 40 | 60 | 2h | RBC velocity | Intestine | ↑ |  |
|  | Wang | 1991 | ATP-MgCl2 | 6 | 40 | ND | 4h | Blood flow | Liver | ↑ |  |
|  | Wang | 1991 | Diltiazem | 8 | 40 | 90 | 4h | Blood flow | Liver | ↑ |  |
|  | Wang | 1992 | ATP-MgCl2 | 7 | 40 | 45 | 4h | Blood flow | Kidney | ↑ |  |
|  | Zakaria | 2005 | Vitasol | 8 | 50 | 60 | 2h | Blood flow | Intestine | ↑ |  |
| **COAGULATION** | Wang | 1990 | Heparin | 6-9 | 40 | 90 | 1.5h | Blood flow | Liver | ↑ |  |
|  | Wang | 1994 | Heparin | 6 | 40 | 90 | 5h | Blood flow | Intestine | = |  |
|  |  |  |  |  |  |  |  |  | Kidney | ↑ |  |
|  |  |  |  |  |  |  |  |  | Liver | = |  |
|  |  |  |  |  |  |  |  |  | Spleen | = |  |
|  | Wang | 1994 | Chemically modified heparin | 6 | 40 | 90 | 5h | Blood flow | Intestine | ↑ |  |
|  |  |  |  |  |  |  |  |  | Kidney | ↑ |  |
|  |  |  |  |  |  |  |  |  | Liver | = |  |
|  |  |  |  |  |  |  |  |  | Spleen | ↑ |  |
|  | Wang | 1996 | GM1892 | 6-7 | 40 | 90 | 4h | Blood flow | Intestine | ↑ |  |
|  |  |  |  |  |  |  |  |  | Kidney | ↑ |  |
|  |  |  |  |  |  |  |  |  | Liver | ↑ |  |
|  |  |  |  |  |  |  |  |  | Spleen | ↑ |  |
|  | Zaets | 2011 | Recombinant human FXIII A2 subunit | 8 | 30 | 90 | 3h | Blood flow | Liver | ↑ |  |
|  |  |  |  |  |  |  |  |  | Muscle | ↑ |  |

Summary of results: ‘↑’ indicates an increase and ‘=’ no difference in blood flow or red blood cell (RBC) velocity, as reported by the study’s authors, following treatment compared to the corresponding control group receiving solely fluid resuscitation. ND; not determined

**Table S5.** Summary of study findings regarding therapeutics targeting the complement system or systemic inflammation, endothelial barrier modulators and hormones

|  | **First author** | **Year** | **Name of drug** | **Group size (n)** | **Depth of HS (mmHg)** | **Duration HS (min)** | **Measurement time point after resuscitation** | **Outcome** | **Organ** | **Summary** |  |
| --- | --- | --- | --- | --- | --- | --- | --- | --- | --- | --- | --- |
| **COMPLEMENT SYSTEM** | Fruchterman | 1998 | sCR1 (recombinant human soluble complement receptor1) | 6 | 45 | 60 | 2.5h | Blood flow | Intestine | ↑ | |
|  | Horstick | 2001 | C1-INH (C1-esterase inhibitor) | 6 | 30 | 60 | 4h | RBC velocity | Intestine | = | |
| **ENDOTHELIAL BARRIER MODULATORS** | Liu | 2014 | Platelet-derived growth factor | 8 | 40 | 120 | 2h | Blood flow | Kidney | ↑ |  |
|  |  |  |  |  |  |  |  |  | Liver | ↑ |  |
|  | Trieu | 2018 | Vasculotide | 7 | 30 | 60 | 1h | Perfusion | Cremaster muscle | ↑ |  |
| **HORMONES** |  |  |  |  |  |  |  |  | Intestine | ↑ |  |
|  | Ba | 2001 | Flutamide | 6 | 40 | 90 | 21h | Blood flow | Kidney | ↑ |  |
|  |  |  |  |  |  |  |  |  | Liver | ↑ |  |
|  | Ba | 2005 | 17beta-estradiol (E2) | ND | 40 | 90 | 2h | Blood flow | Intestine | = |  |
|  | Bertuglia | 2004 | Adrenocorticotropic-stimulating hormone (ACTH (1-2+)) | 5-10 | 30 | 45 | 1.5h | Blood flow | Cheek pouch | ↑ |  |
|  | Bowen | 1979 | Methylprednisolone sodium succinate | 6 | 40 | 210 | 2h | Blood flow | Spleen | ↓ |  |
|  | Hergenroder | 1989 | Dopamine | 7-11 | 30 | ND | 1h | Blood flow | Brain | = |  |
|  | Ida | 2018 | Terlipressin | 26 | 40 | 30 | 2h | Functional vessel density | Brain | = |  |
|  | Pinilla | 1977 | Methylprednisolone | 15 | 30 | 90 | 1h | Blood flow | Muscle | = |  |
|  | Remmers | 1997 | Flutamide | 10-11 | 40 | 45 | 20h | Blood flow | Liver | ↑ |  |
|  | Vollmer | 2017 | Melatonin | 5 | 50 | 60 | 1h | Blood flow | Stomach | = |  |
| **INFLAMMATION** | Bauer | 1995 | IL-1ra | 8 | 40 | 90 | 5h | Blood flow | Liver | = |  |
|  | Marzi | 1993 | Dexamethasone | 4-6 | 40 | 60 | 0.5h | Perfusion | Liver | = |  |
|  | Marzi | 1993 | Ibuprofen | 4-6 | 40 | 60 | 0.5h | Perfusion | Liver | = |  |
|  | Marzi | 1993 | MK886 | 4-6 | 40 | 60 | 0.5h | Perfusion | Liver | = |  |
|  | Marzi | 1995 | Anti-TNF monoclonal antibody (TN3) | 6 | 40 | 45 | 5h | Blood flow | Liver | = |  |
|  | Wallace | 1997 | Aspirin | 5 | 25 | 15 | ND | Blood flow | Stomach | = |  |

Summary of results: ‘↑’ indicates an increase and ‘=’ no difference and ‘↓’ a decrease in blood flow, red blood cell (RBC) velocity, functional vessel density or perfusion as reported by the study’s authors, following treatment compared to the corresponding control group receiving solely fluid resuscitation. ND; not determined

**Table S6.** Summary of study findings regarding vasoactive agents

|  | **First author** | **Year** | **Name of drug** | **Group size (n)** | **Depth of HS (mmHg)** | **Duration HS (min)** | **Measurement time point after resuscitation** | **Outcome** | **Organ** | **Summary** |
| --- | --- | --- | --- | --- | --- | --- | --- | --- | --- | --- |
| **VASOACTIVE AGENTS** | Angele | 1999 | L-arginine | 6-8 | 40 | 90 | 1h | Blood flow | Spleen | ↑ |
|  | Ba | 2005 | BQ-123 | ND | 40 | 90 | 2h | Blood flow | Intestine | ↑ |
|  | Bauer | 2004 | S-nitroso-albumin (S-NO-HSA) | 8 | 40 | 60 | 5h | Blood flow | Liver | ↑ |
|  |  |  |  |  |  |  | 24h |  | Liver | ↑ |
|  | Bini | 2018 | Vasopressin | 10 | 40 | 60 | 1.5h | Blood flow | Intestine | ↓ |
|  | Brouse | 2015 | RRx-001 | ND | 45 | 60 | 1.5h | Blood flow | Skin | ↑ |
|  | Cabrales | 2009 | Sodium nitrite | 6 | 40 | 60 | 1.5h | Blood flow | Skin | = |
|  | Hergenroder | 1989 | dipyridamole | 6-11 | 30 | ND | 1h | Blood flow | Brain | = |
|  | Hergenroder | 1989 | RA 642 | 8-11 | 30 | ND | 1h | Blood flow | Brain | ↑ |
|  | Lima | 2012 | Noradrenaline | 10 | 60 | 60 | 1h | RBC velocity | Skin | = |
|  | Lima | 2012 | Vasopressin | 10 | 60 | 60 | 1h | RBC velocity | Skin | = |
|  | Lima | 2019 | Levosimendan | 10 | 40 | 60 | 1.5h | RBC velocity | Skin | = |
|  | Lima | 2019 | Norepinephrine | 10 | 40 | 60 | 1.5h | RBC velocity | Skin | = |
|  | Lima | 2019 | Sodium nitroprusside | 10 | 40 | 60 | 1.5h | RBC velocity | Skin | = |
|  | Michida | 1994 | BQ-123 | 5 | 40 | 60 | 45min | Blood flow | Stomach | ↑ |
|  |  |  |  |  |  |  |  |  | Adrenal | ↑ |
|  |  |  |  |  |  |  |  |  | Heart | ↓ |
|  |  |  |  |  |  |  |  |  | Intestine | ↓ |
|  |  |  |  |  |  |  |  |  | Kidney | ↓ |
|  | Olsen | 1969 | Metaraminol - concious | 10 | 50 | ND | ND | Blood flow | Liver | = |
|  |  |  |  |  |  |  |  |  | Lung | = |
|  |  |  |  |  |  |  |  |  | Muscle | = |
|  |  |  |  |  |  |  |  |  | Skin | ↓ |
|  |  |  |  |  |  |  |  |  | Stomach | = |
|  |  |  |  |  |  |  |  |  | Adrenal | ↓ |
|  |  |  |  |  |  |  |  |  | Heart | ↑ |
|  |  |  |  |  |  |  |  |  | Intestine | ↓ |
|  |  |  |  |  |  |  |  |  | Kidney | ↓ |
|  | Olsen | 1969 | Metaraminol - unconcious | 10 | 50 | ND | ND | Blood flow | Liver | ↓ |
|  |  |  |  |  |  |  |  |  | Lung | = |
|  |  |  |  |  |  |  |  |  | Muscle | ↓ |
|  |  |  |  |  |  |  |  |  | Skin | = |
|  |  |  |  |  |  |  |  |  | Stomach | ↓ |
|  | Schmidt | 2006 | Dihydralazine | 6 | 40 | 60 | 5h | Blood flow | Liver | = |
|  | Schmidt | 2006 | Urapidil | 6 | 40 | 60 | 5h | Blood flow | Liver | = |
|  | Szabo | 2004 | ETR-p1/f1 peptide | 8-10 | 40 | 60 | 4h | RBC velocity | Intestine | = |
|  | Wallace | 1997 | NCX-4016 | 5 | 25 | 15 | ND | Blood flow | Stomach | = |

Summary of results: ‘↑’ indicates an increase and ‘=’ no difference and ‘↓’ a decrease in blood flow or red blood cell (RBC) velocity, as reported by the study’s authors, following treatment compared to the corresponding control group receiving solely fluid resuscitation. ND; not determined

**Table S7.** Summary of study findings regarding other therapeutics targeting homeostasis

|  | **First author** | **Year** | **Name of drug** | **Group size (n)** | **Depth of HS (mmHg)** | **Duration HS (min)** | **Measurement time point after resuscitation** | **Outcome** | **Organ** | **Summary** |
| --- | --- | --- | --- | --- | --- | --- | --- | --- | --- | --- |
| **OTHERS** | Kubulus | 2008 | Hemin arginate | 8 | 35 | 60 | 2h | Blood flow | Liver | ↑ |
|  | Roesner | 2006 | 5-AIQ | 7 | 40 | 60 | 5h | Perfusion | Liver | ↑ |
|  | Torres Filho | 2017 | Adenosine-lidocaine-magnesium (ALM) | 9 | 50 | 60 | 1.5h | Blood flow | Cremaster muscle | = |
|  | Torres Filho | 2017 | Beta-hydroxybutyrate + melatonin (HB/M) | 9 | 50 | 60 | 1.5h | Blood flow | Cremaster muscle | = |
|  | Torres Filho | 2017 | Poloxamer -188 | 9 | 50 | 60 | 1.5h | Blood flow | Cremaster muscle | = |
|  | Wang | 1996 | GM6001 | 6-7 | 40 | 90 | 4h | Blood flow | Intestine | ↑ |
|  |  |  |  |  |  |  |  |  | Kidney | ↑ |
|  |  |  |  |  |  |  |  |  | Liver | ↑ |
|  |  |  |  |  |  |  |  |  | Spleen | = |

Summary of results: ‘↑’ indicates an increase and ‘=’ no difference in blood flow or perfusion, as reported by the study’s authors, following treatment compared to the corresponding control group receiving solely fluid resuscitation.

**Figure S1**. Summary of risk of bias


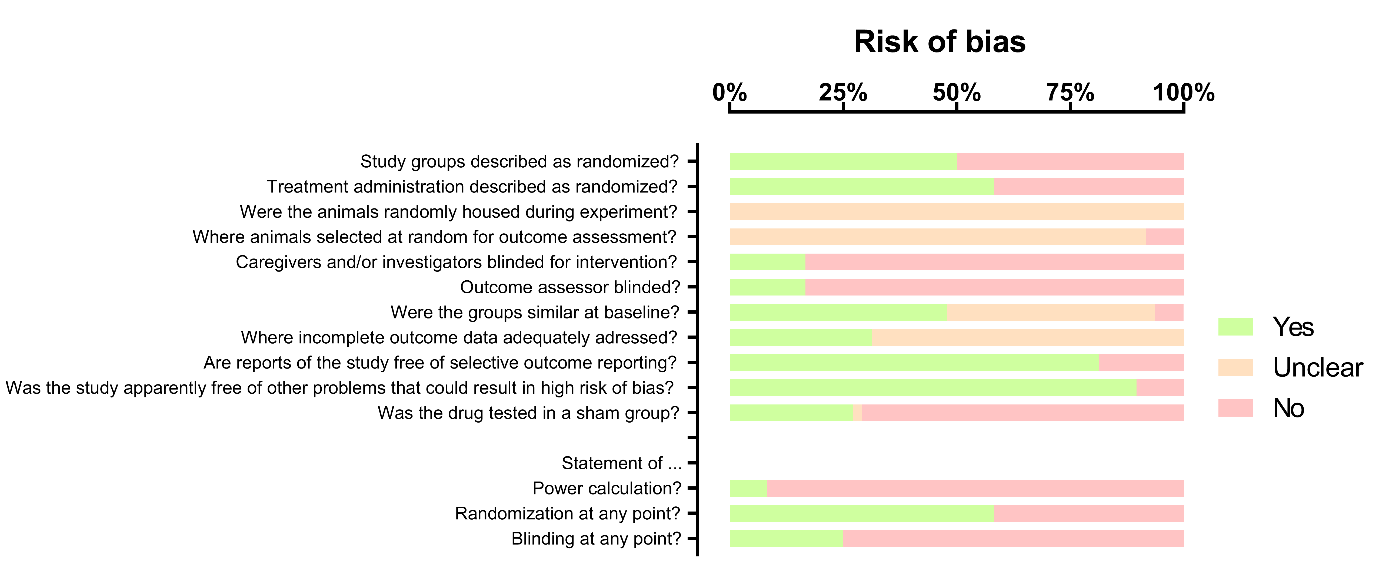


**Figure S2**. Funnel plot of all included studies


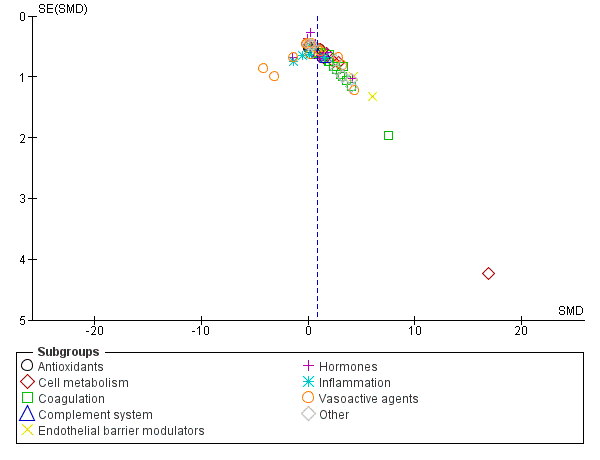

Supplement: Supplementary file 1 — Supinfo [file MICC-27-e12650-s001.docx]
